# Investigation into the genetics of fetal congenital lymphatic anomalies

**DOI:** 10.1002/pd.6345

**Published:** 2023-04-03

**Authors:** Daniella Rogerson, Anna Alkelai, Jessica Giordano, Madhulatha Pantrangi, Meng-Chang Hsiao, Chia-Ling Nhan-Chang, Joshua E. Motelow, Vimla Aggarwal, David Goldstein, Ron Wapner, Carrie J. Shawber

**Affiliations:** 1 Columbia University Vagelos College of Physicians and Surgeons, New York, New York, USA; 2 Department of Obstetrics and Gynecology, Columbia University Vagelos College of Physicians and Surgeons, New York, New York, USA; 3 Institute for Genomic Medicine, Columbia University Irving Medical Center, New York, New York, USA; 4 Department of Pathology and Cell Biology, Columbia University Vagelos College of Physicians and Surgeons, New York, New York, USA; 5 Department of Pediatrics, Columbia University Vagelos College of Physicians andSurgeons, New York, New York, USA; 6 Department of Surgery, Columbia University Vagelos College of Physicians and Surgeons, New York, New York, USA

## Abstract

**Objective:**

Congenital lymphatic anomalies (LAs) arise due to defects in lymphatic development and often present in utero as pleural effusion, chylothorax, nuchal and soft tissue edema, ascites, or hydrops. Many LAs are caused by single nucleotide variants, which are not detected on routine prenatal testing.

**Methods:**

Demographic data were compared between two subcohorts, those with clinically significant fetal edema (CSFE) and isolated fetal edema. A targeted variant analysis of LA genes was performed using American College of Medical Genetics criteria on whole exome sequencing (WES) data generated for 71 fetal edema cases who remained undiagnosed after standard workup.

**Results:**

CSFE cases had poor outcomes, including preterm delivery, demise, and maternal preeclampsia. Pathogenic and likely pathogenic variants were identified in 7% (5/71) of cases, including variants in RASopathy genes, *RASA1, SOS1, PTPN11,* and a novel *PIEZO1* variant. Variants of uncertain significance (VOUS) were identified in 45% (32/71) of cases. In CSFEs, VOUS were found in *CELSR1, EPHB4*, *TIE1*, *PIEZO1*, *ITGA9*, RASopathy genes, *SOS1, SOS2*, and *RAF1*.

**Conclusions:**

WES identified pathogenic and likely pathogenic variants and VOUS in LA genes in 51% of fetal edema cases, supporting WES and expanded hydrops panels in cases of idiopathic fetal hydrops and fluid collections.

## 1 | INTRODUCTION

Congenital lymphatic anomalies (LAs) often arise in the fetal period due to defects in lymphatic development. LAs can present as cystic lymphatic malformations (CLMs), primary lymphedema, and complex lymphatic anomaly, which include generalized lymphatic anomalies (GLAs), Kaposiform Lymphangiomatosis (KLA), Gorham-Stout Disease (GS), central conducting lymphatic anomaly (CCLA), and generalized lymphatic dysplasia (GLD).^1,2^ Increased nuchal translucency (NT) can occur secondary to aberrant lymphatic development, and many cystic hygromas (CHs) are fetal cervicofacial CLMs.^3–6^ Fetal CLMs also present in axillary and other soft tissues.^7,8^ Complex LAs are diffuse multifocal lymphatic hyperplasias that can involve the lungs, intestines, liver, spleen, soft tissues and bone.^9,10^ CCLAs are due to defects in the abdominal and thoracic lymphatic collecting ducts, resulting in backflow into the lymphatic capillary networks of the liver, intestines, and lungs.^9–11^ Complex LAs can cause fetal pleural/pericardial effusion, ascites, chylothorax, soft tissue edema, and nonimmune hydrops.^6,9,12–16^ Primary lymphedema is marked by the peripheral accumulation of the lymph in the soft tissues of extremities and genitalia.^1,12,14,17,18^ Overall, between 5% and 15% of cases of fetal nonimmune hydrops are suggested to develop due to lymphatic defects.^12–14^

LAs result from both germline autosomal dominant and recessive variants with varying degrees of penetrance as well as somatic variants.^1^ Lymphedema arises from inherited and de novo germline loss of function (LOF) variants in lymphangiogenic genes, *ANGPT2, CCBE1, CELSR1, FLT4, FOXC2, GATA2, GJA1, GJC2, HGF, KIF11, IKBKG, SOX18, TIE1, PTPN14*, and *VEGFC*.^1^ Variants in *CALCRL, EPHB4, PIEZO1*, and *ITGA9* are associated with prenatal CCLA and GLD and precipitate nonimmune hydrops and chylothorax.^19–22^ LAs are often present as part of RASopathy syndromes with variants in the genes *PTPN11, RAF1, NRAS, KRAS, HRAS, RASA1, RIT1, SOS1*, and *SOS2.* LAs are also found as part of complex syndromes due to chromosomal abnormalities or variants in *PTEN*, *TSC1*, *TSC2*, and *AKT1*.^1,2,12,17^ Lastly, somatic variants in *PIK3CA, NRAS*, and *KRAS* have been described in CLMs and GLAs, which require genetic analysis of affected tissues.^1,23–26^

The ability to differentiate fetal LAs from other etiologies of hydrops and edema relies on genetic diagnosis, but the full scope of the genetic variants that contribute to LAs remains yet to be discovered. Thus, predicting outcomes of fetal edema remains challenging in the euploid, structurally normal fetus. LAs are often due to small nucleotide variants which are not detected by standard karyotyping and microarray. Diagnosis relies upon further testing via either targeted gene panels or whole exome sequencing (WES). Current genetic panels only query a subset of LA genes and the variants tested vary between clinical laboratories.^27^ Thus, genetic panels are not comprehensive. We queried for variants in LA genes using WES of parental-fetal trios from a cohort of idiopathic fetal fluid collection and hydrops, including increased NT, CH, cystic lesions, pleural effusion, chylothorax, ascites, and lymphedema. Our targeted analysis identified pathogenic and likely pathogenic variants and variants of uncertain significance (VOUS) in fetal edema cases. Many of these were not identified in a prior untargeted study.^28^ As predicting pregnancy outcomes in euploid fetal edema cases remains challenging,^29,30^ demographic data were compared between two subcohorts, clinically significant fetal edema (CSFE) versus isolated fetal edema (IFE). A better understanding of the LA variants which contribute to fetal hydrops and demographic factors associated with poor outcomes will aid in diagnosis and counseling in the prenatal period.

## 2 | METHODS

### 2.1 | Participants and study design

Fetal anomaly cases enrolled in a WES study at Columbia University (IRB:AAAO8009) between 2015 and 2019 were reviewed. Pathogenic WES findings for most fetal anomaly cases were previously published.^28^ In the current study, cases of pathologic fluid collection ascertained by prenatal ultrasound were identified, including increased NT (≥3.5 mm), CH, increased nuchal fold (≥6.0 mm), pleural effusion, chylothorax, ascites, skin and soft tissue edema, cystic structures consistent with CLM, and hydrops, defined as two or more fluid collections. Prenatal chylothorax was defined distinct from pleural effusion if prenatal thoracentesis was performed and ≥80% lymphocytes noted. To determine if there are any factors associated with the edema phenotype, fetal edema cases were subdivided into two subcohorts: cases with CSFE defined as requiring prenatal intervention, precipitating fetal outcome, or the effusion persisting postnatally and cases of IFE that resolved within the study period. Demographics and outcomes were compared between the two subcohorts. Outcomes included live birth, intrauterine fetal demise (IUFD), neonatal demise, spontaneous abortion (SAB), and iatrogenic abortion (IAB). Pregnancy end was defined as the end of pregnancy due to any of these outcomes. Known noncongenital lymphatic etiologies of fetal edema were excluded, including anemia preceding edema, immune hydrops, infection, skeletal-thoracic structural anomaly, twin cord/placental cord abnormality, fetal akinesia sequence, aneuploidy, and complex syndrome with unclear etiology of edema. Cases with concurrent gastrointestinal, genitourinary, and cardiac anomalies were not excluded as a number of complex syndromes copresent with LAs.^1,17^ Cases with diagnostic karyotype or microarray or limited data were excluded.

### 2.2 | Clinical data, whole exome sequencing, and variant calling

Medical records and imaging were reviewed, and samples underwent WES at the Institute for Genomic Medicine with the NimblegenSeqCap EZ V2.0/3.0, SeqCap EZ HGSC VCRome, or xGenExome Research Panel v1.0 kits with the Illumina NovaSeq platform. Reads were aligned using human reference GRCh37 with Illumina DRAGEN Bio-IT Platform, and duplicates were marked with Picard (Broad Institute). Variants were called using Genome Analysis Toolkit (GATK) Best Practices v3.6 and annotated with ClinEff and Analysis Tool for Annotated Variants (ATAV) (in-house Analysis Tool for Annotated Variants).^31^ For LA variants with low alternative allele frequencies (≤0.4 for heterozygous and ≤0.9 for homozygous), Integrative Genomic Viewer (IGV), ATAV, and population data were reviewed by a multidisciplinary group, and variants likely to be artifacts were excluded. A modified version of our well-established diagnostic trio analysis framework was used (https://redmine.igm.cumc.columbia.edu/projects/atav/wiki/Diagnostic_Analysis_Workflow).^28,32–34^ Geographic ancestry was determined using a neural network pre-trained on samples with known ancestry, which generated probability estimates for each of the six groups (European, African, Latino, East Asian, South Asian, and Middle Eastern). An exome-based relatedness screen was performed employing KING to calculate pairwise kinship coefficients. Maternity/paternity was confirmed for de novo variants.

The primary endpoint was identification of pathogenic and likely pathogenic variants in LA genes and the identification of unique demographic factors between CSFE and IFE cases. The secondary endpoint was the identification of VOUS in LA genes. LA genes for targeted analysis were determined by review of the basic science and clinical literature and those documented in Online Mendelian Inheritance in Man (Table S1).^1,17^ All candidate genetic variants were classified by American College of Medical Genetics and Genomics (ACMG) and Association for Molecular Pathology criteria informed by ClinGen.^35^ Variants were reviewed by an interdisciplinary team including an expert in LAs and three clinical geneticists. ACMG VOUS among CSFE were determined to have potential clinical significance, given the strong gene–phenotype correlation of examined LA genes and absence of other diagnostic findings. CSFE variants were confirmed by Sanger sequencing except for Fetal0047F for which genomic DNA was not available. ACMG VOUS in the IFE subcohort are reported in Table S2.

### 2.3 | Statistical analysis

Data were analyzed with Prism9. Primary and secondary outcomes are described as percentages. For demographic data, categorical variables are described as percentages and proportions and compared between groups with Fisher’s exact test and Chi-square test. Continuous variables are documented as mean values with interquartile ranges and compared between groups using an unpaired *t*-test or Welch’s *t*-test for data with standard deviation greater than twofold in difference. Normal distribution was examined by cross-checking *t*-test statistics with Mann–Whitney test statistics and histograms. A *p* < 0.05 was considered significant.

## 3 | RESULTS

### 3.1 | Study participants

**FIGURE 1:**
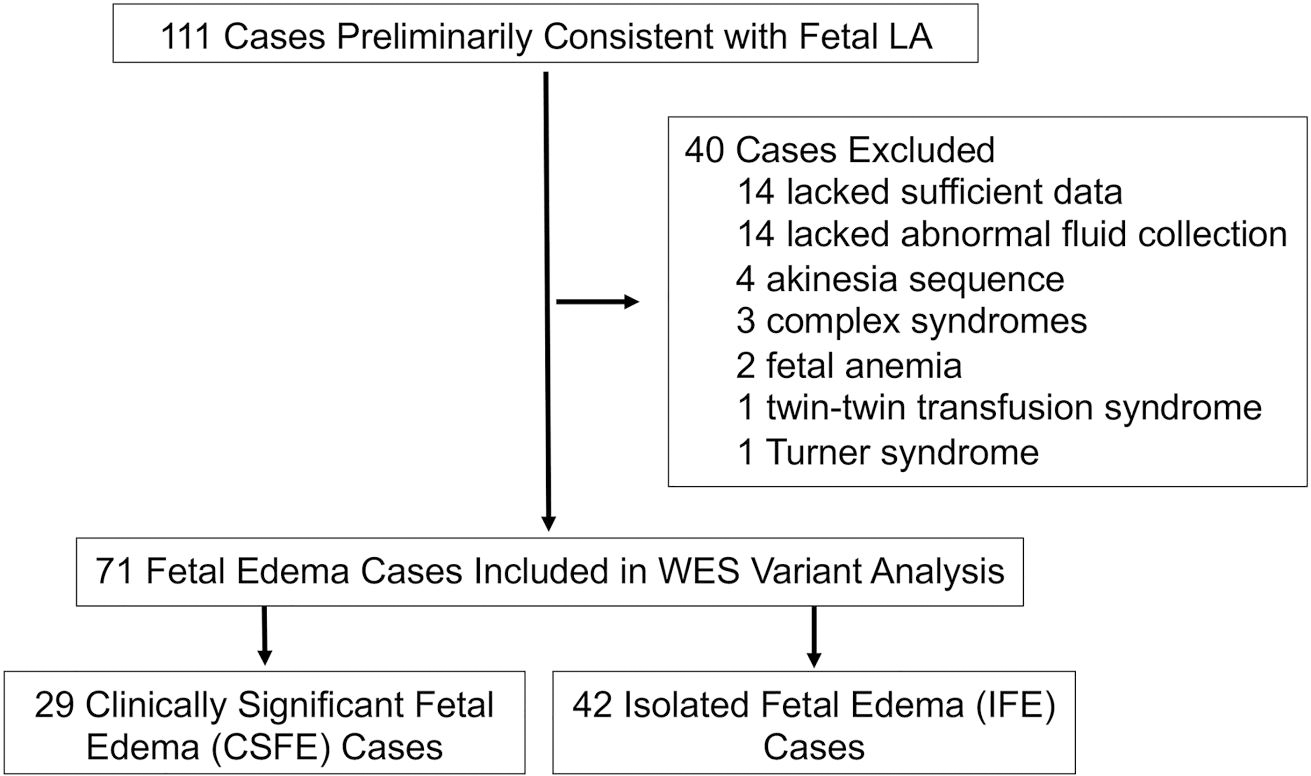
Flow chart of inclusion and exclusion criteria as applied to fetal edema cases.

**FIGURE 2:**
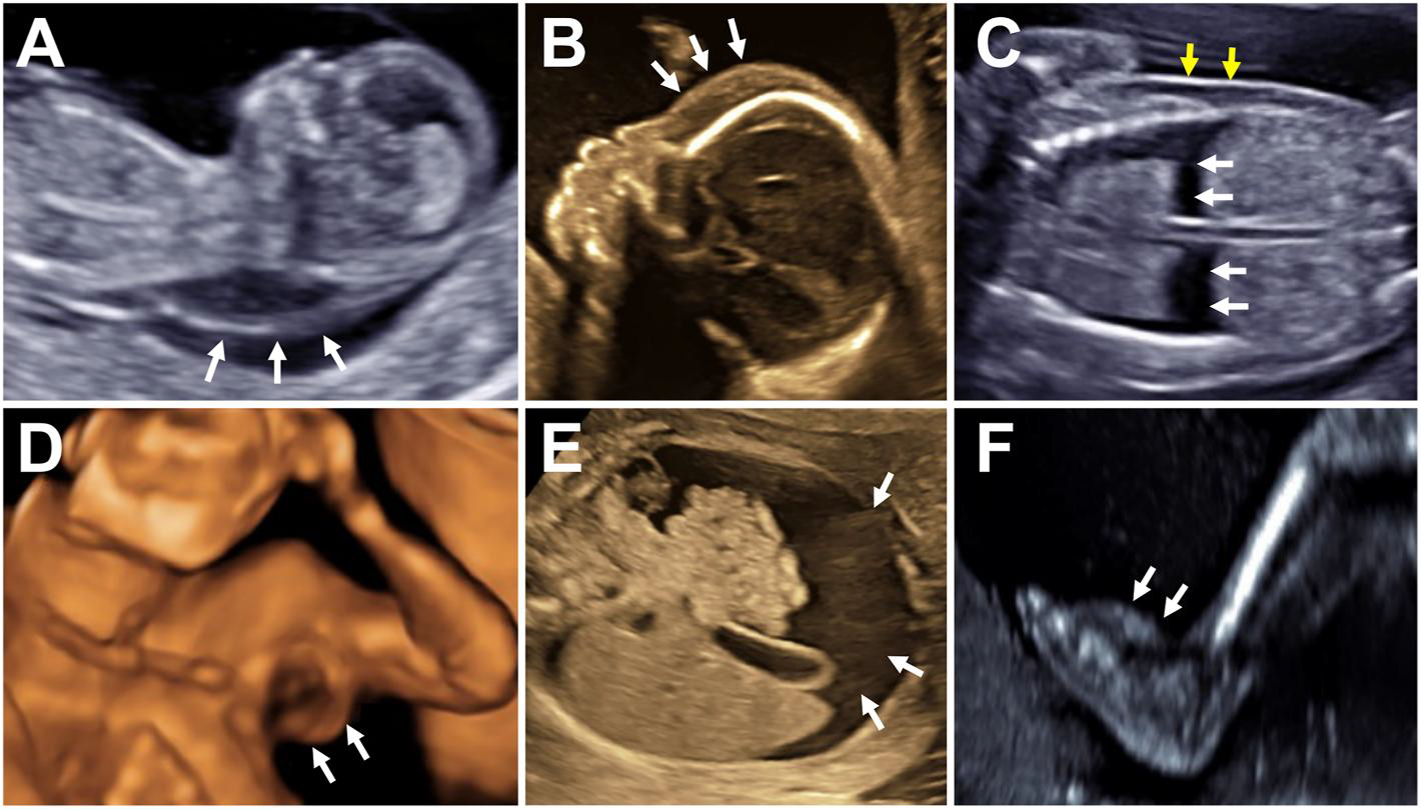
Spectrum of fetal LAs on prenatal ultrasound. (A) Cystic hygroma, 12w (white arrows). (B) Scalp soft tissue edema, 21w (white arrows). (C) Hydrops with bilateral pleural effusion (white arrows) and trunk soft tissue edema (yellow arrows), 19w. (D) 3D ultrasound of an axillary cystic lymphatic malformation, 20w (white arrows). (E) Ascites, 26w (white arrows). (F) Pedal lymphedema, 22w (white arrows). w, weeks’ gestation.

Review of parental-fetal trios identified 111 fetal edema cases, 40 of which were excluded and 71 cases of abnormal fetal fluid collection were examined (Figure 1). Twenty-nine cases displayed CSFE defined as requiring prenatal intervention, precipitating fetal outcome, or the effusion persisting postnatally (Figure 2), while 42 cases were notable for IFE, which required no intervention and resolved within the study period. The CSFE subcohort was compared to the IFE subcohort to determine if there were characteristics unique to the two subcohorts (Table 1). In both subcohorts, over 70% of mothers had ancestry other than European. There was no difference in maternal/paternal age, nulliparity, history of anomaly, fetal sex, microarray finding, or maternal hypertension, diabetes, and BMI. CSFEs were diagnosed at a mean gestation age (GA) of 18 weeks, while IFEs were diagnosed at 11 weeks. The majority 69% (20/29) of CSFEs were hydropic, while 93% (39/42) IFEs presented with isolated nuchal edema. Pregnancy outcomes were significantly different between the two subcohorts. In the CSFEs, 31% (9/29) were live born, 41% (12/29) underwent IAB, 17% (5/29) experienced IUFD, 7% (2/29) neonatal demise, and 3% (1/29) underwent SAB. In the IFEs, 95% (40/42) were live born and 4% (2/42) underwent IAB. Live born CSFEs were delivered at a mean GA of 35 weeks versus 38 for IFEs. Mean GA at pregnancy end for CSFEs was 29 versus 37 weeks for IFEs. CSFEs trended toward having concurrent structural anomaly. Occurrence of preeclampsia with severe features or mirror syndrome occurred in 24% (5/21) of CSFEs versus 2.48% (2/41) of IFEs; 3.45% (1/28) of women with CSFEs used assisted reproductive technology, while 21% (9/42) did among IFEs.

Further subanalysis of anomalies, which presented specifically in the first trimester in CSFEs (*N* = 11) and IFEs (*N* = 40), illustrated that most factors were similar between the two groups (Table S3). However, CSFEs which presented in the first trimester more often had multiple fluid collections; 82% (9/11) of these CSFEs had persistent/worsening edema or another structural anomaly on subsequent scans, while 18% (2/11) appeared structurally normal with resolution of fetal edema but had persistent nuchal edema and dysmorphic features after delivery. In contrast, only 23% (9/40) of IFEs were found to have a concurrent structural anomaly (CNS, renal, skeletal, and cardiac). Occurrence of preeclampsia was 22% (2/9) in the first-trimester CSFEs and 5% (2/40) in IFEs. Overall, pregnancy outcomes remained significantly different between the first-trimester CSFEs and IFEs.

### 3.2 | Pathogenic and likely pathogenic variants in LA genes

Pathogenic and likely pathogenic variants were identified in 17% (5/29) of CSFEs (Table 2). Though not significant, diagnostic yield was lower for CSFE cases with isolated nuchal edema (0%; 0/9) than for cases with multiple fluid collections (25%; 5/20). Pathogenic and likely pathogenic variants were more common, though not significant, in cases with concurrent structural abnormality than without (23%; 3/13 vs. 13%; 2/15). No pathogenic or likely pathogenic variants were identified in IFEs.

Pathogenic and likely pathogenic variants included four genes linked to RASopathies, syndromes that often present with LAs.^1^ Three were heterozygous missense variants in Noonan syndrome genes, *PTPN11* and *SOS1*. A c.417G>C variant in *PTPN11* was identified in two CSFEs: one de novo and one inherited from a mother with Noonan syndrome. A *SOS1* c.1132A>G variant was inherited from a father with Noonan syndrome. The fourth RASopathy variant was a c.2446_2447delGA variant in *RASA1* predicted to cause LOF, which was inherited maternally, though no maternal phenotype was documented by the treating physician. We also identified a novel homozygous stop gained (LOF) variant c.565C>T in the GLD gene *PIEZO1* inherited from consanguineous parents. These findings demonstrate that RASopathies can present as/with LAs and prenatal WES can aid in diagnosing GLD.

### 3.3 | Variants of uncertain significance in LA genes

VOUS in LA genes were identified in 38% (11/29) of CSFEs with 6.9% (2/29) of these carrying VOUS in multiple LA genes (Tables 3 and 4). Similar to pathogenic and likely pathogenic variants, heterozygous VOUS were identified in RASopathy genes (*SOS1, SOS2, and RAF1*), inherited from unaffected parents. Novel VOUS were identified in two cases, a *SOS2* c.1223A>G variant and a *SOS1* c.421A>G variant. A previously reported *RAF1* c.1895_1918delAGGATATCAATGCTTGCACGCTGA VOUS was identified that falls outside the commonly affected domains.^36^

VOUS in genes associated with GLA, primary lymphedema, and dehydrated hereditary stomatocytosis (DHS) with or without perinatal edema were identified in nine cases. Two novel heterozygous missense variants inherited from unaffected parents were found in *EPHB4*, c.250G>A, and c.2957A>G. One of these, Fetal0045F, is notable for carrying a pathogenic *RERE* LOF variant as previously reported.^28,37^ In two cases, novel heterozygous missense variants, inherited from an unaffected parent, c.2888C>T and c.929A>G, were identified in the lymphedema gene *TIE1*.^38^ A novel inherited heterozygous missense variant c.5767G>A was detected in the lymphedema gene, *CELSR1*.

For *PIEZO1*, a novel missense VOUS, c.3208C>T, was identified that was homozygous in the setting of maternal uniparental disomy, while five heterozygous missense variants were identified and inherited from unaffected mothers. One of these cases, Fetal0510F, also carried a pathogenic LOF *GLMN* variant, a gene associated with glomuvenous malformation, which was diagnosed postnatally. In one CSFE case, three *PIEZO1* VOUS that have been previously described were identified in *cis*: c.5290G>C, c.2423G>A, and c.2344G>A.

In 17% (5/29) of CSFEs, a variant was detected for an autosomal recessive disorder consistent with the phenotype, but a second variant was not identified: *ITGA9*, *CCBE1*, and *PTPN14*. Unlike *CCBE1* and *PTPN14,* the inheritance pattern for *ITGA9* variants is not well established; thus, heterozygous VOUS may be of interest (Table 4). We identified novel missense *ITGA9* VOUS inherited from unaffected parents, c.130_131delGCinsAA, c.680C>T, and c.1360G>A.

Heterozygous missense VOUS were identified in 45% (19/42) of IFEs (*ANGPT2, CELSR1, FLT4, HGF, PIEZO1, PTEN, PTPN14, RIT1, TIE1*, and *TSC2*) (Table S2). *PIEZO1*, *CELSR1*, and *TIE1* VOUS in the IFE subcohort were distinct from the CSFE subcohort. Multiple variants were found in 9.5% (4/42) of IFEs.

## 4 | DISCUSSION

Our targeted analysis of LA genes in 71 cases of idiopathic fetal edema identified pathogenic variants in 7% (5/71), and VOUS in 45% (32/71), for an overall yield of 51% (37/71). In CSFEs, pathogenic and likely pathogenic variants were identified in 17% (5/29) of cases. The CSFE subcohort had a slightly lower diagnostic yield than another publication with pathogenic and likely pathogenic variants identified in 29% of nonimmune hydrops cases due to all causes.^30^ The lower yield may be due to our targeted gene approach, as 7% (2/29) of CSFEs had diagnostic variants in non-LA genes (*RERE* and *GLMN*).^28^ Unlike CSFEs, pathogenic and likely pathogenic variants were not identified in IFEs. In CSFE, pathogenic and likely pathogenic variants were identified in fetal hydrops cases, but not those with isolated nuchal edema. A recent WES study had a similarly low yield in cases of isolated nuchal edema (1.8%) versus cases with structural abnormality or hydrops at presentation (22%) or later in pregnancy (32%).^29^ VOUS in LA genes were identified in 45% (13/29) of CSFEs and 45% (19/42) of IFEs; 38% (11/29) of CSFEs and 55% (23/43) of IFEs had no genetic findings, which may be due to several factors. Not all genes that contribute to LAs have been identified and were not included in our analyses. Secondly, somatic mutations often cause LAs, and germline WES will not detect these variants as it requires sequencing of affected tissues.^9^ Lastly, it is likely that some of these fetal edema cases were not secondary to LAs.

Four pathogenic and likely pathogenic variants were in RASopathy genes. RASopathies are heterogenous, overlapping disorders due to hyperactivating variants in the RAS/mitogen-activated protein kinase (MAPK) signaling cascade.^36^ RASopathy variants are a common etiology of hydrops and nuchal edema.^29,30^ In this study, CSFEs with RASopathy variants were all hydropic with pleural effusions and ascites and either with skin edema or polyhydramnios, similar to a recently described RASopathy cohort.^39^ CSFEs with VOUS in RASopathy genes also had hydrops and pleural effusions. Three pathogenic RASopathy variants (*PTPN11, SOS1*) were consistent with Noonan syndrome, an autosomal dominant syndrome of craniofacial and cardiac anomalies often accompanied by prenatal nuchal edema, chylothorax, and hydrops, as well as postnatal lymphedema, GLA, and chylothorax.^1,17,36,40^ The last likely pathogenic RASopathy variant was a frameshift truncation variant in *RASA1*. *RASA1* LOF variants are associated with CM-AVM as a complex vascular anomaly that often concurrently presents with LAs.^1,41,42^ No features of CM-AVM were documented in this case though IUFD occurred and CM-AVM are rarely diagnosed prenatally.^43^ If CM-AVM was absent, this case may represent an expansion of the *RASA1* genotype–phenotype association, which is supported by a *RASA1* deletion variant recently identified in a case of fetal hydrops without CM-AVM.^44^

Analysis of CSFEs identified both likely pathogenic and VOUS variants in *PIEZO1*. Common features among this group included nuchal edema and pleural effusions that were similar to phenotypes observed in recently described *PIEZO1* cases.^39^ Homozygous or compound heterozygous variants in *PIEZO1* are associated with GLAs and marked fetal hydrops, while heterozygous variants are associated with DHS with or without perinatal edema.^19,30,45–49^ We identified a novel homozygous *PIEZO1* missense VOUS in a fetal case of marked hydrops, where neonatal demise occurred on day of life one due to respiratory distress secondary to chylothorax. This variant is absent in large population databases, and there is a strong genotype-phenotype correlation. Additionally, heterozygous VOUS in *PIEZO1* were identified in three cases. In one case of CH and dysmorphic features, three variants were identified in *cis*, two of which, c.2423G>A and c.2344G>A, had previously been identified in *cis* among several family members with DHS with perinatal lymphedema, and in one case, adult lymphedema.^49,50^ Though these variants are individually common in population databases, whether their occurrence in combination is rare enough to be associated with human disease remains unknown. While the other two heterozygous *PIEZO1* VOUS are rare in the general population, *PIEZO1* is a highly polymorphic gene, and functional studies and further clinical data are needed to classify their role in DHS and GLD.

The mechanism of DHS-related perinatal edema is unknown. Though DHS causes mild hemolytic anemia, significant fetal anemia has not been described in these cases and thus likely not the precipitant.^30,48,49^ DHS-associated perinatal edema may be lymphatic in etiology as evidenced by cases of CH, chylothorax, and adult onset lymphedema in the setting of heterozygous *PIEZO1* variants.^30,48,49^ Conversely, homozygous LOF and compound heterozygous missense variants in *PIEZO1* associated with GLD may have DHS like erythrocyte abnormalities though these are not well characterized.^19,48,51^ We identified a novel likely pathogenic homozygous LOF variant in *PIEZO1* in a case of fetal hydrops that subsequently developed significant anemia of unknown etiology. Though transfusion improved anemia, the hydrops was unresolved and IUFD occurred. Given the timeline of hydrops preceding the onset of fetal anemia, anemia was not felt to be the precipitant. Whether this anemia was precipitated by *PIEZO1* variant, which would be a novel presentation, remains unknown as neither fetal nor parental erythrocytes were examined.

Our WES analysis of CSFEs identified VOUS in several additional LA genes, *EPHB4*, *TIE1*, and *CELSR1*. Missense kinase inactivating variants in *EPHB4* are associated with an autosomal dominant GLA that presents with hydrops and postnatal lymphedema.^17,20^ Though the *EPHB4* VOUS we identified falls outside the kinase domain, a likely pathogenic frameshift truncating variant outside the kinase domain has been seen in another case of hydrops.^20,29^ Heterozygous *TIE1* missense variants have been detected in individuals with lower extremity lymphedema, and we describe two novel *TIE1* variants in fetuses with bilateral lower limb edema.^38^ In one fetus with isolated pedal edema, the variant falls within the commonly affected *TIE1* kinase domain.^38^ We also identified a novel missense VOUS in *CELSR1* in a fetus with ascites and hydrocele. While heterozygous *CELSR1* LOF variants are also associated with lower extremity lymphedema, missense variants have been described but have yet to be established as disease causing.^52–54^

A heterozygous *ITGA9* missense variant has been implicated in several cases of recurrent congenital chylothorax and fetal hydrops.^21^ The inheritance pattern for *ITGA9* chylothorax is unknown; though an autosomal recessive mechanism has been proposed, the majority of cases are heterozygous inherited from unaffected parents.^21,55^ Given the reduced penetrance seen in other LAs, this may suggest autosomal dominant inheritance. We identified three heterozygous *ITGA9* missense VOUS in CSFE with pleural effusions that fall outside the protein domain described to be associated with disease (Table 4). One case carried VOUS in *ITGA9, SOS1*, and *TIE1* suggesting a multigenic cause of this fetal LA (Tables 3 and 4).

Small sample size limited our ability to identify predictive factors among first-trimester edema cases as to which would become clinically significant. Consistent with reported poor outcomes for hydropic fetuses, poor outcomes in the CSFE cases included preterm delivery, IUFD, neonatal demise, and maternal preeclampsia.^16,30^ These findings highlight the importance of early detection and management. Prompt clinical recognition of fetal edema followed by genetic diagnosis of LA could allow for intervention for both maternal and fetal benefit.^16^ Emerging therapies include maternal propranolol for congenital chylothorax, as well as the use of trametinib, a RAS/MAPK inhibitor, in RASopathy cases with significant lymphatic sequelae.^56,57^ Maternal propranolol is also an experimental therapy for fetal CLMs.^58^ Genetic diagnosis of LAs also aids in future pregnancy planning, as evidenced by one CSFE case, where the likely pathogenic *PIEZO1* LOF variant was inherited in a homozygous manner in a subsequent pregnancy.

The major strength of this study is the targeted phenotype–genotype approach, which sheds light on the contribution of LAs to prenatal edema and hydrops and describes novel variants in LA genes. This study highlights a set of LA genes, though not exhaustive, which should be included in clinically available LA and hydrops gene panels to aid in diagnostic workup of idiopathic nonimmune hydrops and in suspected neonatal and pediatric LAs. This targeted gene approaches also allowed for the identification of novel VOUS in LA genes, which were otherwise overlooked in an undifferentiated WES analysis.^28^

Study limitations include the inclusion of non-LA cases in the fetal edema cohort. Although inclusion criteria were strict, fetal hydrops is often a nonspecific finding that may be precipitated by numerous nonlymphatic etiologies, including some structural anomalies that were not excluded due to their overlapping phenotypes with LA syndromes.^16^ This inclusion of non-LA cases in a targeted analysis of LA genes may have lowered the diagnostic yield. Our targeted gene approach also did not detect variants in genes outside our panel, which were found in 7% (2/29) of cases. It is possible that aborted cases could have resolved later and thus were included inappropriately among CSFEs. Conversely, LA phenotype in IFEs may have developed postnatally beyond the period of the study.t

## 5 | CONCLUSION

Targeted analysis of LA genes identified variants in 51% (37/71) of fetal edema cases. This supports the use of next-generation sequencing and expanded LA panels for the prenatal diagnosis and further study of fetal LAs. We describe novel variants in several LA genes, including a likely pathogenic variant in *PIEZO1*, and VOUS *in PIEZO1, ITGA9, CELSR1, EPHB4*, and *TIE1*, as well as novel VOUS in RASopathy genes. Further work examining prenatal LAs and describing LA variants is needed.

## Supplementary Material

Supplemental Table 3

Supplemental Table 2

Supplemental Table 1

## Figures and Tables

**TABLE 1:** Characteristics and outcomes for CSFE versus IFE subcohorts.

Characteristic	Clinically significant fetal edema	Isolated fetal edema	Test statistic
Mean maternal age—year (*N*, IQR, Std Dev)^†^	31 (28, 27–35, 4.76)	33 (42, 31–37, 5.24)	*p* = 0.2048
Mean paternal age—year (*N*, IQR, Std Dev)^†^	33 (28, 27–39, 8.40)	36 (42, 31–39, 7.30)	*p* = 0.1618
Nulliparous—no (*N*, %)°	14 (28, 48.28)	18 (42, 42.86)	*p* = 0.8087
Use of ART—no (*N*, %)°	1 (28, 3.45)	9 (42, 21.43)	***p* = 0.0401**
Mean GA at diagnosis—week (*N*, IQR, Std Dev)‡	18 (23, 12–21, 5.77)	11 (40, 11–12, 1.53)	***p* < 0.0001**
Microarray finding—no (*N*, %)°	5 (29, 17.24)	2 (41, 4.88)	*p* = 0.1175
Prior pregnancy anomaly—no (*N*, %)°	3 (29, 10.34)	4 (42, 9.52)	*p* > 0.9999
Parents′ consanguineous—no (*N*, %)°	1 (29, 3.45)	1 (42, 2.38)	*p* > 0.9999
Prenatal phenotype—no (*N*, %)^#^			***p* < 0.0001**
Isolated nuchal edema	6 (29, 20.69)	39 (42, 92.86)	
Isolated non-nuchal fluid collection	3 (29, 10.34)	1 (42, 2.38)	
≥2 abnormal fetal fluid collections	20 (29, 68.97)	2 (42, 4.76)	
Concurrent structural anomaly—no (*N*, %)°	13 (28, 46.43)	10 (42, 23.80)	*p* = 0.0693
Fetal sex—no (*N*, %)°			*p >* 0.9999
Female	12 (29, 41.38)	17 (42, 40.48)	
Male	17 (29, 58.62)	25 (42, 59.52)	
Maternal ancestry—no (*N*, %)°			*p* = 0.7783
African	2 (29, 6.90)	5 (42, 11.90)	
Caucasian	6 (29, 20.69)	11 (42, 26.19)	
East Asian	2 (29, 6.90)	3 (42, 7.14)	
Hispanic	8 (29, 27.59)	15 (42, 35.71)	
Middle Eastern	6 (29, 20.69)	3 (42, 7.14)	
Mixed	3 (29, 10.34)	1 (42, 2.38)	
South Asian	2 (29, 6.90)	4 (42, 9.52)	
Maternal chr. hypertension—no (*N*, %)°	1 (27, 3.70)	3 (42, 7.14)	*p* > 0.9999
Maternal diabetes—no (*N*, %)°	0 (28, 0.00)	3 (42, 7.14)	*p* = 0.2696
Mean maternal BMI—(*N*, IQR, Std Dev)^†^	25.14 (16, 22.08–26.08, 4.93)	28.26 (40, 22.77–32.15, 6.99)	*p* = 0.1097
Pregnancy outcome—no (*N*, %)°			***p* < 0.0001**
Live birth	9 (29, 31.03)	40 (42, 95.24)	
IAB	12 (29, 41.38)	2 (42, 4.76)	
IUFD	5 (29, 17.24)	0 (42, 0.00)	
Neonatal demise	2 (29, 6.90)	0 (42, 0.00)	
SAB	1 (29, 3.45)	0 (42, 0.00)	
Mean GA at live delivery—week (*N*, IQR, Std Dev)^†^	35 (10, 33–38, 3.37)	38 (40, 37–39, 1.97)	***p* = 0.0004**
Mean GA at pregnancy end—week (*N*, IQR, Std Dev)^†^	29 (22, 23–36, 7.78)	37 (42, 36–39, 4.94)	***p* < 0.0001**
Maternal preeclampsia spectrum—no (*N*, %)°	5 (21, 23.81)	2 (41, 2.38)	***p* = 0.0387**

*Note*: Percentages may not total 100 due to rounding to the nearest two‐digit decimal place. Categorical variables are compared with Fisher's exact test ° and Chi‐square test^#^.

Continuous variables are compared between groups using an unpaired *t*-test^†^ or Welch's *t*-test^‡^ for data with standard deviation greater than twofold in difference. Ancestry was as determined by genetic profiling.

Diabetes was type I or II, nongestational. Hypertension preceded pregnancy (chronic). Concurrent structural anomalies include those not related to abnormal fluid collection and diagnosed prenatally. Test statistics for fetal outcome and for ancestry are comparing live birth versus adverse outcome and Caucasian versus non-Caucasian, respectively. Pregnancy end includes all outcomes. All cases in this cohort were preeclampsia with severe features, or mirror syndrome though charts were reviewed for gestational hypertension, preeclampsia, preeclampsia with severe features, superimposed preeclampsia, eclampsia, and mirror syndrome. Significant statistical values are in bold.

Abbreviations: ART, assisted reproductive technology; CSFE, clinically significant fetal edema; GA, gestational age; IAB, iatrogenic abortion; IFE, isolated fetal edema; IQR, interquartile range; IUFD, intrauterine fetal demise (after 20w); SAB, spontaneous abortion (prior to 20w); Std Dev, standard deviation.

**TABLE 2:** Pathogenic and likely pathogenic variants in congenital lymphatic anomaly genes.

Case	Prenatal phenotype	Postnatal phenotype	Concurrent structural anomaly^a^	Fetal or neonatal intervention^b^/outcome	Gene/RefSeq ID	Genomic coordinate (GRCh37/hgl9)/nucleotide/protein alteration	Molecular consequence	OMIM genetic disorder/ MIM No.	Inheritance/ zygosity	Novel or PMID/ CLINVAR Ac. No	ACMG classifi cation
Fetai0164F	Pleural effusion, ascites, hydrocele, skin and scalp edema, and polyhydramnios	NA	None	None/IUFD	RASA 1/NM_002890.3	5–86674312-AAG-Ac.2446_2447delGA p.Asp816Leufs*13	Frameshift	Capillary malformation-arteriovenous malformation 1/608354	Maternal/heterozygous	30712878	Likely pathogenic PVS1, PM2
Feta10222F	Increased NT, scalp, skin and significant nuchal fold and shoulder edema, bilateral pleural effusion, ascites, and polyhydramnios	Bilateral pleural effusion, ascites, anasarca, respira tory di stress, dysmorphic features, webbed neck, pulmonic valve stenosis, and atrial septal defect	None	Neonatal ventilation, chest tu be placement and pulmonic valve repair complicated by prolonged postsurgical chylothorax/ live born	SOS V NM_005633.4	2–39251221-T-C c.H32A>G p.Thr378Ala	Missense	Noonan syndrome 4/610733	Paternal/ heterozygous	21387466	Pathogenic PS1, PS4 (moderate), PM2, PM6 and, PP1
Fetai0302F	Left > right pleural effusion, ascites, and polyhydramnios	Dysmorphic features, redundant nuchal skin folds, undescended testes, and respiratory distress	Bilateral ventricular hypertrophy, atypical or absent cavum septum pellucidum, and Blake's pouch cyst	Serial thoracentesis (2x) followed by thoracic shunt placement/ live born	PTPN11/NM_002834.5	12–112891083- G-C c.417G>C p.Glul39Asp	Missense	Noonan syndrome 1/163950	Maternal/ heterozygous	28363362	Pathogenic PS1, PS2, PS3, PS4 (moderate), PM2, PP1, PP2, and PP3
Feta10405F	Scalp, total body skin, nuchal fold edema, bilateral pleural effusion, ascites, and pericardial effusion	NA	Cardlomegaly, agenesis of the ductus venosus, placentomegaly, abnormal profile, and enlarged tongue, anemia	Percutaneous umbilical blood sampling with transfusion/ IUFD	PIEZ01/ NM_001142864.4	16–88805045- G- A c.565C>T p.Argl89*	Nonsense	Lymphatic malformation 6/616843	Both parents/ homozygous	Novel	Likely pathogenic PVS1, PM2
Fetal0485F	Bilateral pleural effusion, ascites, and unilateral shoulder edema	NA	Dilated left ventricle	None/IUFD	PTPN11/NM_002834.5	12–112891083- G-C c.417G>C p.Glul39Asp	Missense	Noonan syndrome 1/163950	De Novo/ heterozygous	28363362	Pathogenic PS1, PS2, PS3, PS4 (moderate), PM2, PP1, PP2, and PP3

Abbreviations: CH, Cystic hygroma; IAB, iatrogenic abortion; IUFD, Intrauterine fetal demise; NT, Nuchal translucency; OMIM, Online Mendelian Inheritance in Man.

a Concurrent structural anomalies are those that presented prenatally.

b Intervention does not include iatrogenic delivery for non‐reassuring fetal monitoring or maternal indication.

**TABLE 3:** VOUS in congenital lymphatic anomaly genes.

Case	Prenatal phenotype	Postnatal phenotype	Concurrent structural anomaly^a^	Fetal or neonatal intervention^b^/outcome	Gene/RefSeq ID	Genomic coordinate (GRCh37/hgl9)/nucleotide/protein alteration	Molecular consequence	OMIM genetic disorder/MIM No.	Inheritance/zygosity	Novel or PMID/CLINVAR Ac. No	ACMG classification
Fetal0032F	CH, enlarged nuchal fold, total body anasarca with edema especially of the scalp and trunk with a small pleural effusion	NA	Cardiomegaly, small stomach, two vessel cords, skeletal growth delay, enlarged kidneys	None/IUFD	*EPHB4*/NM_004444.5	7–100421427-C-T c.250G>A p.Val84lle	Missense	Lymphatic malformation 7/617300	Paternal/heterozygous	Novel	vous PM2 and BP4
Fetal0038F	Right chylothorax, pericardial effusion	None	None	Serial thoracentesis (5x), maternal propranolol/liveborn	*SOS2*/NM_006939.4	14–50626778-T-Cc.l223A>G p.His408Arg	Missense	Noonan syndrome 9/616559	Maternal/heterozygous	Novel	VOUS PM2, BP4
Fetal0045F	CH	Dysmorphic features, right undescended testicle, hypertonia, spasticity, and redundant nuchal skin	None	None/liveborn	*EPHB4*/NM_ 004444.5	7–100401090-T-C c.2957A>G p.Gln986Arg	Missense	Lymphatic malformation 7/617300	Paternal/heterozygous	Novel	VOUS PM2, BP4, BP5
Fetal0047F	Increased NT, bilateral pedal edema especially at the anterior and plantar surfaces	NA	None	None/IAB	*TIE1*/NM_001253357.2	1–43783709-C-T c.2888C>T p.Ala963Val	Missense	Lymphatic malformation 11/619401	Maternal/heterozygous	Novel	VOUS PM2, BP4
Fetal0052F	Left chylothorax	NA	None	Serial thoracentesis (2x), maternal propranolol/IAB	*PIEZOl*/NM_001142864.4	16–88808727-G-C c.264C>G p.Asp88Glu	Missense	Dehydrated hereditary stomatocytosis with or without perinatal edema/194380	Maternal/heterozygous	Novel	VOUS PM2, BP4
Fetal0151F	Pericardial effusion, ascites, and hydrocele	Cardiomyopathy, dysplastic mitral value, respiratory distress, neonatal heart failure, dysmorphic features, and ascites	Cardiomyopathy and valvular dysfunction	Maternal digoxin, neonatal mechanical ventilation, and mitral valvuloplasty/neonatal demise	*CELSR1*/NM_014246.4	22–46792578-C-T c.5767G>A p.Vall923Met	Missense	Lymphatic malformation 9/619319	Maternal/heterozygous	Novel	VOUS PM2, BP1
Fetal0231F	CH	Dysmorphic features, redundant nuchal skin, fifth toe clinodactyly, and bilateral ventriculomegaly	Bilateral ventriculomegaly	None/Liveborn	*PIEZ01*/NM_001142864.4	16–88788059-C-G c.5290G>C p.Glul764Gln	Missense	Dehydrated hereditary stomatocytosis with or without perinatal edema/194380	Maternal/heterozygous	VCV001049973.1	vous PM2
					*PIEZ01*/NM_001142864.4	16–88800060-C-T c.2423G>A p.Arg808Gln	Missense	Dehydrated hereditary stomatocytosis with or without perinatal edema/194380	Maternal/heterozygous	30655378	VOUS PP1, BS1
					*PIEZOl*/NM_001142864.4	16–88800139-C-T c.2344G>A p.Gly782Ser	Missense	Dehydrated hereditary stomatocytosis with or without perinatal edema/194380	Maternal/heterozygous	30655378	VOUSPP1, BS1
Fetal0310F	Increased nuchal fold, bilateral pleural effusion, total body skin edema, ascites, pericardial effusion, and polyhydramnios	Macrocephaly, anasarca, bilateral pleural effusion, hypospadias, and respiratory distress	None	Neonatal mechanical ventilation and bilateral chest tube placement/neonatal demise	*PIEZOl*/NM_001142864.4	16–88794058-G-A c.3208C>T p.Argl070Cys	Missense	Lymphatic malformation 6/616843	Maternal uniparental disomy/homozygous	Novel	VOUS PM2, PP3
Fetal0399F	Ascites, facial edema especially scalp and periorbital, lower extremity edema, and right pleural effusion	NA	None	None/IAB	*SOSl*/NM_005633.4	2–39283932-T-C c.421A>G p.llel41Val	Missense	Noonan syndrome 4/610733	Paternal/heterozygous	Novel	VOUS PM2, BP4
					*TIE1*/NM_001253357.2	1-43773482-A-G c.929A>G p.His310Arg	Missense	Lymphatic malformation 11/619401	Maternal/ heterozygous	Novel	VOUS PM2, BP4
Fetal0445F	Pleural effusion, other undescribed second abnormal fluid collection	NA	Unknown	Unknown/IAB	*RAF1*/NM_002880.4	3–12626041-GTCAGCGTGCAAGCATTGATATCCTGc.1895_1918delAGGATATCAATGCTTGCACGCTGA p.Glu632_Thr640delinsAla	Disruptive inframe deletion	Noonan syndrome 5/611553	Paternal/heterozygous	VCV00092899 2.1	VOUS PM2, PM4
Feta10510F	Bilateral chylothorax, ascites	Pleural effusion, respiratory distress, and right lower quadra nt venous-lymphatic malformation	None	Serial thoracentesis (8x), chest tube placement, ventilation, propranolol, diuresis/live born	*PIEZ01*/NM_001142864.4	16–88798811-G-C c.2923C>G p.Leu975Val	Missense	Dehyd rated hereditary stomatocytosis with or without perinatal edema/194380	Maternal/heterozygous	Novel	vous PM3, BP5

Abbreviations: CH, Cystic hygroma; IAB, iatrogenic abortion; IUFD, Intrauterine fetal demise; NT, Nuchal translucency; OMIM, Online Mendelian Inheritance in Man.

a Concurrent structural anomalies are those that presented prenatally.

b Intervention does not include iatrogenic delivery for non‐reassuring fetal monitoring or maternal indication.

**TABLE 4:** Heterozygous VOUS in genes of uncertain inheritance.

Case	Prenatal phenotype	Postnatal phenotype	Concurrent structural anomaly^a^	Fetal or neonatal intervention^b^/outcome	Gene/RefSeq ID	Genomic coordinate (GRCh37/hgl9)/nucleotide/protein alteration	Molecular consequence	OMIM genetic disorder/MIM No.	Inheritance/zygosity	Novel or PMID/CLINVAR Ac. No	ACMG classification
Fetal0399F	Ascites, facial edema especially scalp and periorbital, lower extremity edema, and right pleural effusion	NA	None	None/IAB	*ITGA9*/NM_002207.3	3–37493995-GC-AA c.l30_131delGCinsAA p.Ala44Asn	Missense	Congenital Chylothorax/NA	Paternal/heterozygous	Novel	VOUS PM1, PM2
Fetal0403F	Bilateral pleural effusion, thickened nuchal fold, skin edema, ascites, and pericardial effusion	NA	None	None/IAB	*ITGA9*/NM_002207.3	3–37544736-C-T c.680C>T p.Thr227Met	Missense	Congenital Chylothorax/NA	Paternal/heterozygous	Novel	VOUS PM2, PP3
Fetal0523F	CH, skin edema of the chest, and pleural effusion	NA	None	None/SAB	*ITGA9*/NM_002207.3	3–37567549-G-A c.l360G>A p.Val454Met	Missense	Congenital Chylothorax/NA	Maternal/heterozygous	Novel	VOUS PM2, PP3

Abbreviations: CH, Cystic hygroma; IAB, iatrogenic abortion; OMIM, Online Mendelian Inheritance in Man; SAB, spontaneous abortion.

a Concurrent structural anomalies are those that presented prenatally.

b Intervention does not include iatrogenic delivery for non‐reassuring fetal monitoring or maternal indication.

## Data Availability

The primary study submits all genotype and phenotype data to the database of Genotypes and Phenotypes (dbGaP) at the completion of the study as per agreement with the NIH. A copy of the data sharing certificate is available for documentation.

## Key points

### What’s known on this topic?

- Congenital lymphatic anomalies (LAs) arise due to defects in lymphatic development.
- Variants in several genes are known to cause LAs though many of variants remain unknown.
- The full contribution of LA genes to fetal hydrops and nuchal edema remains unknown.

### What does the study add?

- The study identifies novel likely pathogenic variants and variants of uncertain significance via whole exome sequencing and analysis of genes associated with LAs.
- Identified variants may improve diagnosis of fetal LAs among otherwise idiopathic fetal hydrops cases.
