# Supplementary material for: Investigation into the genetics of fetal congenital lymphatic anomalies
Source: Prenat Diagn. Author manuscript; Available in PMC 2023 Jul 10. (PMC10330091; doi:10.1002/pd.6345)
Supplement: Supplemental Table 3 [file NIHMS1889580-supplement-Supplemental_Table_3.docx]

| **Supplemental Table 3. Characteristics and Outcomes for Cases with First Trimester Nuchal Edema** | | | |
| --- | --- | --- | --- |
| **Characteristic** | **Clinically Significant Fetal Edema** | **Isolated Fetal Edema** | **Test Statistic** |
| Mean maternal age – year (N, IQR, Std Dev)^†^ | 30 (11, 26-34, 4.83) | 32 (40, 31-37, 5.36) | p = 0.1167 |
| Mean paternal age – year (N, IQR, Std Dev)^†^ | 32 (11, 26-40, 7.82) | 35 (35, 32-39, 6.93) | p = 0.1846 |
| Nulliparous – no (N, %)**°** | 6 (11, 54.55) | 18 (40, 45.00) | p = 0.7358 |
| Use of ART – no (N, %)**°** | 0 (11, 0.00) | 9 (40, 22.5) | p = 0.1767 |
| Mean GA at diagnosis – week (N, IQR, Std Dev) ^†^ | 11 (8, 11-12, 1.31) | 11 (38, 11-12, 0.90) | p = 0.4089 |
| Microarray finding – no (N, %)**°** | 2 (11, 18.18) | 2 (39, 5.13) | p = 0.2063 |
| Prior pregnancy anomaly – no (N, %)**°** | 0 (11. 0.00) | 4 (40, 10.00) | p = 0.5651 |
| Parents consanguineous – no (N, %)**°** | 0 (11, 0.00) | 1 (40, 2.50) | p > 0.9999 |
| Prenatal phenotype – no (N, %)**°** |  |  | **p = 0.0002** |
| Isolated nuchal edema | 5 (11, 45.45) | 39 (40, 97.5) |  |
| ≥2 abnormal fetal fluid collections | 6 (11, 54.54) | 1 (40, 0.2.5) |  |
| Concurrent structural anomaly – no (N, %)**°** | 5 (11, 45.45) | 9 (40, 22.5) | p = 0.1484 |
| Mean NT/CH Size, mm (N, IQR, Std Dev) ^†^ | 5.45 (10, 3.80-7.48, 2.06) | 4.68 (39, 3.60-5.60, 1.35) | p = 0.1595 |
| Fetal sex – no (N, %)**°** |  |  | p = 0.4967 |
| Female | 6 (11, 54.55) | 16 (40, 40.00) |  |
| Male | 5 (11, 45.45) | 24 (40, 60.00) |  |
| Maternal ancestry – no (N, %)**°** |  |  | p = 0.7063 |
| African | 0 (11, 0.00) | 5 (40, 12.50) |  |
| Caucasian | 2 (11, 18.18) | 11 (40, 27.50) |  |
| East Asian | 1 (11, 9.09) | 3 (40, 7.50) |  |
| Hispanic | 4 (11, 36.36) | 13 (40, 32.50) |  |
| Middle Eastern | 2 (11, 18.18) | 3 (40, 7.50) |  |
| Mixed | 2 (11, 18.18) | 1 (40, 2.50) |  |
| South Asian | 0 (11, 0.00) | 4 (40, 10.00) |  |
| Maternal chr. hypertension – no (N, %)**°** | 1 (10, 10.00) | 3 (40, 7.50) | p > 0.9999 |
| Maternal diabetes – no (N, %)**°** | 0 (11, 0.00) | 3 (40, 7.50) | p > 0.9999 |
| Mean maternal BMI – (N, IQR, Std Dev) ^‡^ | 25.18 (7, 24.33-25.76, 1.74) | 28.29 (39, 22.66-31.95, 7.05) | p = 0.4504 |
| Pregnancy outcome – no (N, %)**°** |  |  | **p = 0.0006** |
| Livebirth | 5 (11, 45.45) | 38 (40, 95.00) |  |
| IAB | 4 (11, 36.36) | 2 (40, 5.00) |  |
| IUFD | 1 (11, 9.09) | 0 (40, 0.00) |  |
| Neonatal Demise | 0 (11, 0.00) | 0 (40, 0.00) |  |
| SAB | 1 (11, 9.09) | 0 (40, 0.00) |  |
| Mean GA at live delivery – week (N, IQR, Std Dev)­^†^ | 37 (11, 35-38, 2.00) | 38 (40, 36-39, 2.01) | p = 0.2777 |
| Mean GA at pregnancy end, all outcomes – week (N, IQR, Std Dev) ^‡^ | 28 (9, 18-38, 10.32) | 37 (40, 36-39, 5.05) | **p = 0.0408** |
| Maternal preeclampsia spectrum – no (N, %)**°** | 2 (9, 22.22) | 2 (40, 5.00) | p = 0.1490 |
| Percentages may not total 100 due to rounding to the nearest two-digit decimal place. Categorical variables are compared with Fisher’s exact test**°** and Chi-square test**^#^**. Continuous variables are compared between groups using an unpaired t-test^†^ or Welch’s t-test^‡^ for data with standard deviation greater than two-fold in difference Ancestry was as determined by genetic profiling. Diabetes was type I or II, non-gestational. Hypertension preceded pregnancy (chronic). Concurrent structural anomalies include all those not related to abnormal fluid collection and diagnosed prenatally. Test statistics for fetal outcome and for ancestry are comparing livebirth versus adverse outcome and Caucasian versus non-Caucasian, respectively. Pregnancy end includes all outcomes. All cases in this cohort were preeclampsia with severe features or mirror syndrome though charts were reviewed for gestational hypertension, preeclampsia, preeclampsia with severe features, superimposed preeclampsia, eclampsia, and mirror syndrome. IAB – iatrogenic abortion. IUFD - intrauterine fetal demise (after 20w). SAB - spontaneous abortion (prior to 20w). ART - assisted reproductive technology. GA- gestational age. IQR - interquartile range, Std Dev - standard deviation | | | |
