# Supplementary material for: Investigation into the genetics of fetal congenital lymphatic anomalies
Source: Prenat Diagn. Author manuscript; Available in PMC 2023 Jul 10. (PMC10330091; doi:10.1002/pd.6345)
Supplement: Supplemental Table 2 [file NIHMS1889580-supplement-Supplemental_Table_2.docx]

| **Supplemental Table 2. VOUS in Congenital Lymphatic Anomaly Genes in IFE cohort** | | | | | | | | | | |  |
| --- | --- | --- | --- | --- | --- | --- | --- | --- | --- | --- | --- |
| **Case** | **Prenatal Imaging Phenotype** | **Postnatal Phenotype** | **Concurrent Structural Anomaly**^†^ | **Outcome** | **Gene/**  **RefSeq Id** | **Genomic Coordinate**  **(GRCh37/hg19)/**  **Nucleotide /Protein Alteration** | **Molecular Consequence** | **OMIM Genetic Disorder/ MIM No.** | **Inheritance/ Zygosity** | **Novel or PMID/ CLINVAR Ac. No** | **ACMG Classification** |
| **Fetal0006F** | Increased NT | None | None | Liveborn | *PIEZO1*/ NM_001142864.4 | 16-88782507-C-T  c.100G>A  p.Gly34Ser | Missense | Dehydrated hereditary stomatocytosis with or without perinatal edema/ 194380 | Maternal/ Heterozygous | Novel | VOUS  PM2, BP4 |
| **Fetal0009F** | CH | None | None | Liveborn | *PIEZO1*/  NM_001142864.4 | 16-88786822-T-C  c.5920A>G  p.Ile1974Val | Missense | Dehydrated hereditary stomatocytosis with or without perinatal edema/ 194380 | Maternal/  Heterozygous | Novel | VOUS  PM2, BP4 |
|  |  |  |  |  | *CELSR1*/  NM_014246.4 | 22-46930600-C-T  c.2468G>A  p.Arg823His | Missense | Lymphatic malformation 9/619319 | Paternal/  Heterozygous | Novel | VOUS  PM2 |
| **Fetal0040F** | Increased NT | None | None | Liveborn | *TSC2*/ NM_000548.5 | 16-2106700-G-C  c.704G>C  p.Ser235Thr | Missense | Tuberous sclerosis- 2/613254 | Maternal/  Heterozygous | VCV000486692.3 | VOUS  PM2 |
| **Fetal0042F** | Increased NT | None | None | Liveborn | *FLT4*/  NM_182925.5 | 5-180057229-C-T  c.509G>A  p.Arg170His | Missense | Lymphatic malformation 1/153100 | Paternal/  Heterozygous | Novel | VOUS  PM2, BP4 |
| **Fetal0043F** | CH | Small anterior fontanelle | None | Liveborn | *TIE1*/  NM_001253357.2 | 1-43774584-C-T  c.1085C>T  p.Thr362Met | Missense | Lymphatic malformation 11/619401 | Paternal/  Heterozygous | Novel | VOUS  PM2, BP4 |
| **Fetal0054F** | Increased NT | None | None | Liveborn | *HGF/*  NM_000601.6 | 7-81336624-C-T  c.1598G>A  p.Arg533Gln | Missense | Lymphedema/ NA | Paternal/  Heterozygous | Novel | VOUS  PM2 |
| **Fetal0060F** | CH | Penile webbing | None | Liveborn | *PIEZO1*/  NM_001142864.4 | 16-88790362-A-G  c.4252T>C  p.Tyr1418His | Missense | Dehydrated hereditary stomatocytosis with or without perinatal edema/ 194380 | Maternal/  Heterozygous | VCV000618279.7 | VOUS  PM2, PP3 |
| **Fetal0115F** | CH | None | None | Liveborn | *PIEZO1*/  NM_001142864.4 | 16-88793468-A-G  c.3434T>C  p.Val1145Ala | Missense | Dehydrated hereditary stomatocytosis with or without perinatal edema/ 194380 | Maternal/  Heterozygous | VCV000618272.6 | VOUS  PM2 |
|  |  |  |  |  | *PIEZO1*/  NM_001142864.4 | 16-88800949-G-A  c.1998-3C>T  NA | Splice region variant | Dehydrated hereditary stomatocytosis with or without perinatal edema/ 194380 | Maternal/  Heterozygous | Novel | VOUS  PM2, BP4 |
| **Fetal0134F** | Increased NT | None | None | Liveborn | *PTEN*/ NM_000314 | 10-89653851-T-C  10-89653851-T-C  c.149T>C  p.Ile50Thr | Missense | PTEN Hamartoma Tumor Syndrome, 158350 | Maternal/ Heterozygous | 24375884 | VOUS  PM1, PM2, PP3 |
| **Fetal0136F** | Increased NT | Speech and swallow dysfunction, right limb weakness | None | Liveborn | *RIT1*/ NM_006912.6 | 1-155874217-C-T  c.365G>A  p.Arg122His | Missense | Noonan syndrome  8/615355 | Maternal/  Heterozygous | Novel | VOUS  PM2 |
| **Fetal0169F** | CH | None | None | Liveborn | *CELSR1*/  NM_014246.4 | 22-46932139-A-G  c.929T>C  p.Val310Ala | Missense | Lymphatic malformation 9/619319 | Paternal/  Heterozygous | Novel | VOUS  PM2 |
| **Fetal0180F** | Increased NT | None | None | Liveborn | *ANGPT2*/  NM_001147.3 | 8-6385181-G-A  c.461C>T  p.Thr154Met | Missense | Lymphatic malformation 10/619369 | Maternal/  Heterozygous | Novel | VOUS  PM2 |
| **Fetal0204F** | CH | None | None | Liveborn | *CELSR1*/  NM_014246.4 | 22-46787526-G-A  c.6148+4C>T  NA | Splice region variant | Lymphatic malformation 9/619319 | Maternal/  Heterozygous | Novel | VOUS  PM2, BP4 |
| **Fetal0206F** | Increased NT | None | None | Liveborn | *PIEZO1*/ NM_001142864.4 | 16-88783618-T-C  c.6473A>G  p.Lys2158Arg | Missense | Dehydrated hereditary stomatocytosis with or without perinatal edema/ 194380 | Paternal/  Heterozygous | VCV001306254.2 | VOUS  PM2 |
| **Feal0214F** | CH | None | None | Liveborn | *PTPN14*/  NM_005401.5 | 1-214557727-G-A  c.1471C>T  p.Arg491Trp | Missense | Choanal atresia and lymphedema/ 613611 | Maternal/  Heterozygous | Novel | VOUS  PM2 |
| **Fetal0429F** | Increased NT | None | None | Liveborn | *TIE1*/  NM_001253357.2 | 1-43787130-G-A  c.3212G>A  p.Arg1071His | Missense | Lymphatic malformation 11/619401 | Paternal/  Heterozygous | Novel | VOUS  PM2, PP3 |
| **Fetal0467F** | Increased NT | None | None | Liveborn | *TSC2*/ NM_000548.5 | 16-2135264-G-A  c.4603G>A, p.Asp1535Asn | Missense | Tuberous sclerosis-2, 613254 | Paternal/  Heterozygous | VCV000798176.3 | VOUS  PM2, PP3 |
|  |  |  |  |  | *PIEZO1*/ NM_001142864.4 | 16-88789000-G-A  c.4766C>T  p.Thr1589Ile | Missense | Dehydrated hereditary stomatocytosis with or without perinatal edema/ 194380 | Maternal/  Heterozygous | VCV000735052.1 | VOUS  PM2, BP4 |
| **Fetal0516F** | CH | None | None | Liveborn | *HGF/* NM_000601.6 | 7-81355325-C-T  c.1049G>A  p.Arg350Gln | Missense | Lymphedema/ NA | Maternal/  Heterozygous | Novel | VOUS  PM2 |
| **Fetal0538F** | Increase NT | None | None | Liveborn | *PIEZO1*/ NM_001142864.4 | 16-88804377-G-C  c.985C>G  p.Leu329Val | Missense | Dehydrated hereditary stomatocytosis with or without perinatal edema/ 194380 | Maternal/  Heterozygous | VCV000781344.2 | VOUS  PM2, BP4 |
| **^†^**Concurrent structural anomalies are those that presented prenatally. NT – Nuchal translucency. CH – Cystic hygroma | | | | | | | | | | |  |
