# Supplementary material for: Investigation into the genetics of fetal congenital lymphatic anomalies
Source: Prenat Diagn. Author manuscript; Available in PMC 2023 Jul 10. (PMC10330091; doi:10.1002/pd.6345)
Supplement: Supplemental Table 1 [file NIHMS1889580-supplement-Supplemental_Table_1.docx]

| **Supplemental Table 1. LA Associated Genes** | | | |
| --- | --- | --- | --- |
| **Gene** | **LA/Syndrome with LA** | **Inheritance** | **Penetrance/Expressivity** |
| *AKT1* | Proteus Syndrome | Somatic |  |
| *ANGPT2* | Lymphedema | Autosomal dominant | Incomplete penetrance |
| *CALCRL* | Nonimmune lymphatic-related hydrops fetalis | Autosomal recessive |  |
| *CCBE1* | Hennekam lymphangiectasia-lymphedema syndrome | Autosomal recessive | Highly penetrant |
| *CELSR1* | Lymphedema | Autosomal dominant | Incomplete penetrance |
| *EPHB4* | Nonimmune lymphatic-related hydrops fetalis | Autosomal dominant | Variable expressivity |
| *FLT4* | Milroy disease | Autosomal dominant | Variable expressivity |
| *FOXC2* | Lymphedema distichiasis syndrome | Autosomal dominant | Variable expressivity |
| *GATA2* | Emberger syndrome | Autosomal dominant | Incomplete penetrance |
| *GJA1* | Oculodentodigital Syndrome | Autosomal dominant |  |
| *GJC2* | Lymphedema (Meige disease) | Autosomal dominant |  |
| *HGF* | Lymphedema/lymphangiectasia | Unknown |  |
| *ITGA9* | Chylothorax | Unknown |  |
| *KIF11* | Microcephaly with or without chorioretinopathy, lymphedema, or mental retardation | Autosomal dominant | Variable expressivity, incomplete penetrance |
| *IKBKG (NEMO)* | Anhydrotic ectodermal dysplasia with immunodeficiency, osteopetrosis, and lymphedema | X-linked | Incomplete penetrance |
| *PIEZO1* | Generalized lymphatic dysplasia with nonimmune fetal hydrops | Autosomal recessive |  |
|  | Dehydrated hereditary stomatocytosis with or without perinatal edema | Autosomal dominant |  |
| *PIK3CA* | CLOVES | Somatic |  |
|  | Klippel-Trenaunay-Weber syndrome | Somatic |  |
|  | Common cystic lymphatic malformation | Somatic |  |
|  | Generalized lymphatic anomaly | Somatic |  |
| *PTEN* | Cowden syndrome | Autosomal dominant |  |
| *PTPN11* | Noonan syndrome | Autosomal dominant | Incomplete penetrance |
| *PTPN14* | Choanal atresia and lymphedema | Autosomal recessive |  |
| *RAF1* | Noonan syndrome | Autosomal dominant | Incomplete penetrance |
| *NRAS* | Noonan syndrome | Autosomal dominant |  |
|  | Generalized lymphatic anomaly | Somatic |  |
| *KRAS* | Noonan syndrome | Autosomal dominant | Incomplete penetrance |
|  | Generalized lymphatic anomaly | Somatic |  |
| *HRAS* | Costello syndrome | Autosomal dominant | Incomplete penetrance |
| *RASA1* | Noonan syndrome | Autosomal dominant |  |
| *RIT1* | Noonan syndrome | Autosomal dominant | Incomplete penetrance |
| *SOS1* | Noonan syndrome | Autosomal dominant |  |
| *SOS2* | Hypotrichosis-lymphedema-telangiectasia-renal-defect syndrome | Autosomal dominant, autosomal negative | Variable expressivity |
| *SOX18* | Lymphedema | Autosomal dominant | Variable expressivity |
| *TIE1* | Tuberous sclerosis | Autosomal dominant |  |
| *TSC1* | Tuberous sclerosis | Autosomal dominant |  |
| *TSC2* | Lymphangiomatosis | Somatic |  |
| *VEGFC* | Lymphedema | Autosomal dominant |  |
